# Diversity and Composition of the Adult Fecal Microbiome Associated with History of Cesarean Birth or Appendectomy: Analysis of the American Gut Project

**DOI:** 10.1016/j.ebiom.2014.11.004

**Published:** 2014-11-08

**Authors:** James J. Goedert, Xing Hua, Guoqin Yu, Jianxin Shi

**Affiliations:** Division of Cancer Epidemiology and Genetics, National Cancer Institute, National Institutes of Health, Bethesda, MD 20892, USA

**Keywords:** Human microbiome, Feces, Adults, Birth history, Cesarean section, Appendectomy history

## Abstract

**Background:**

Cesarean birth is associated with altered composition of the neonate's microbiota and with increased risk for obesity and other diseases later in life. The mechanisms of these associations, and whether cesarean birth is associated with an altered adult microbiota, are unknown.

**Methods:**

In 1097 adult volunteers without diabetes, inflammatory bowel disease, or recent antibiotic use, fecal microbiome metrics were compared by history of cesarean birth (N = 92) or appendectomy (N = 115). Associations with potential confounders, microbiome alpha diversity, and individual microbial taxa were estimated by logistic regression. Permutation tests assessed differences in microbial composition (beta diversity) based on Jensen–Shannon divergence.

**Findings:**

Cesarean birth history was associated with younger age; appendectomy with older age and higher body mass index. Neither was associated with fecal microbiome alpha diversity. Microbial composition at all taxonomic levels differed significantly with cesarean birth (P ≤ 0.008) but not with appendectomy (P ≥ 0.29). Relative abundance differed nominally for 17 taxa with cesarean birth and for 22 taxa with appendectomy, none of which was significant with adjustment for multiple comparisons.

**Interpretation:**

Adults born by cesarean section appear to have a distinctly different composition of their fecal microbial population. Whether this distinction was acquired during birth, and whether it affects risk of disease during adulthood, are unknown.

**Funding:**

Supported by the Intramural Research Program, National Cancer Institute, National Institutes of Health (Z01-CP-010214).

## Highlights

• Fecal microbiome differed in composition for adults who had been born by cesarean section versus vaginal delivery.
• No difference was found for adults with a history of appendectomy.
• Source of the cesarean-associated microbiome distinction, and possible effects on disease during adulthood, is unknown.

## 1 Introduction

Prenatal and early postnatal exposures and events can affect the entire life course. As one example, cesarean birth has been associated with an increased likelihood of asthma and cardiovascular disease in children (Renz-Polster et al., 2005, Thavagnanam et al., 2008, Friedemann et al., 2012), hypertension in young adults (Horta et al., 2013), and obesity in both children and adults (Pei et al., 2014, Darmasseelane et al., 2014, Blustein et al., 2013, Mueller et al., 2014). While these associations are certainly multifactorial, differences in the gut microbiota could contribute. As well summarized by Arrieta and colleagues, several studies have noted differences in the neonatal fecal microbiota by route of delivery (Arrieta et al., 2014). Using aerobic and anaerobic cultures, Adlerberth et al. found higher abundance of *Escherichia coli* and lower abundance of enterobacteria in 99 vaginally delivered compared 17 cesarean delivered newborns (Adlerberth et al., 2006). Biasucci et al. used PCR amplification of Bifidobacterium species as well as PCR-denaturing gradient gel electrophoresis to find that 23 cesarean-delivered newborns had lower bacterial diversity and an absence of Bifidobacteria compared to 23 vaginally delivered newborns (Biasucci et al., 2008). Among 1032 infants studied at age 1 month, Penders and colleagues used polymerase chain reaction to quantify total bacteria and five bacterial taxa, finding that cesarean birth was associated with higher carriage of *Clostridium difficile* and lower abundance of Bacteroides and Bifidobacteria (Penders et al., 2006), thereby confirming Biasucci et al. (2008). More recently, with comprehensive analysis based on next generation sequencing of 16S rRNA genes, Dominguez-Bello and colleagues reported that route of delivery was associated with differences in the composition of the microbial populations that initially colonized the offspring. Notably, neonates who were born vaginally were colonized by vagina-associated bacteria, whereas those born by cesarean section were initially colonized by skin-associated bacteria (Dominguez-Bello et al., 2010).

Early life alteration of the gut microbiota may have a lasting effect. Trasande et al. observed that exposure to antibiotics up to age 6 months was associated with elevated body mass index (BMI) up to age 7 years (Trasande et al., 2013). In a recently reported murine model, Cox and colleagues observed that prenatal and postnatal exposures to subtherapeutic doses of penicillin resulted in an alteration of the gut microbiota that was transient (Cox et al., 2014). However, the early life exposure to penicillin also caused prolonged metabolic alterations including exacerbated diet-induced obesity (Cox et al., 2014). These observations are consistent with studies in humans showing that the distal gut microbial population may have a major effect on the risks for obesity and malnutrition. Among American adults, the composition of the microbial population in feces is generally altered with obesity, with enrichment by taxa in the phylum Firmicutes (Turnbaugh et al., 2009). Conversely, Malawian infants and young children with Kwashiorkor also have an altered population of fecal microbes, without a clear taxonomic signature but with a disease phenotype that could be transmitted by transplantation of Kwashiorkor feces to gnotobiotic mice (Smith et al., 2013). Likewise, the penicillin-induced obesity phenotype in the mouse could be transferred by fecal transplantation (Cox et al., 2014).

If cesarean delivery has a prolonged effect on the microbiota, this could contribute to the risk for metabolic diseases later in life. Herein, we explored whether the fecal microbiota differs between adults who reported that they were born by cesarean versus vaginal delivery. For comparison, in the same population we looked for differences in the fecal microbiota with history of appendectomy (Guinane et al., 2013, Randal Bollinger et al., 2007). The appendix, particularly its microbial-rich biofilm, has long been postulated to serve as a repository for repopulating the distal gut following an insult such as diarrheal disease or antibiotic exposure (Randal Bollinger et al., 2007). Whether and how this occurs is unknown, particularly in light of a recent observation that surgically removed appendices contained some taxa that are infrequently found in the distal gut (Guinane et al., 2013). In either case, removal of the appendix could result in a persistent alteration of the fecal microbiota.

## 2 Methods

The 16S rRNA V4 region was sequenced by the American Gut Project. The operational taxonomic unit (OTU) table rarefied to 10,000 sequence reads per sample, as well as metadata, was downloaded from the American Gut Project website (https://github.com/biocore/American-Gut/tree/master/data/AG). A current summary is available at http://microbio.me/AmericanGut/static/img/mod1_main.pdf, and details of the OTU picking and taxonomy assignment are available at http://nbviewer.ipython.org/github/biocore/American-Gut/blob/master/ipynb/module2_v1.0.ipynb. Alpha diversity (number of OTUs, Shannon's index, Chao1, Phylogenetic diversity_whole tree), UniFrac distance matrix and relative abundance of different taxa were calculated in the Quantitative Insights Into Microbial Ecology (QIIME) pipeline.

### 2.1 Data Cleaning and Exclusions

In the full data set, there were 1962 samples, 174 phenotypic variables and 3599 taxa with relative abundance. Among those, 1134 samples were left after exclusions [age < 4 years (number removed, 121), sex missing (196), race missing (19), specimen not feces (393), used antibiotic in the past month (131), has diabetes (107) or inflammatory bowel disease (170)]. After that, 37 duplicated samples were also removed. The remaining 1097 samples were used for statistical analysis (1040 for cesarean associations, and 1076 for appendectomy associations).

### 2.2 Covariate Tests

We used logistic regression to test the association between history of cesarean birth (CSECTION in the data set) and the following variables: age, race/ethnicity, sex, geographic region, BMI, weight change, diet type, gluten sensitivity (GLUTEN), lactose intolerance (LACTOSE), asthma, and frequencies of smoking and alcohol consumption. The logistic regression was repeated with history of appendectomy (APPENDIX_REMOVED) as the dependent variable and the same dependent variables.

### 2.3 Alpha Diversity and Individual Taxa Tests

We used logistic regression to test associations of case status (cesarean birth or appendectomy) with richness, standard estimates of alpha diversity (Shannon, Chao 1, inverse Simpson, and phylogenetic distance whole tree indices) and each of the 1949 taxa in the 1097 samples, adjusting for age, sex and race/ethnicity. The Wald test was used to calculate P values as the primary test. Significance after false discovery rate (FDR) adjustment for multiple comparisons was also considered.

### 2.4 Clustering Analysis and Testing

As used previously to define and test associations with clusters of the microbiome in the vagina (Gajer et al., 2012), our primary analysis was based on Jensen–Shannon (J–S) divergence at each taxonomic level from phylum down to species. For K taxa at a given level, let *P*^*i*^ = (*p*_1_^*i*^, ⋯, *p*_*K*_^*i*^) denote the relative abundances for subject *i*. The Kullback–Leibler (K–L) divergence between subject i and j is defined as *D*_*KL*_(*P*^*j*^|*P*^*i*^) = ∑_*k* = 1_^*K*^*p*_*k*_^*i*^ log(*p*_*k*_^*i*^/*p*_*k*_^*j*^) with respect to the distribution of subject *i*. Since the K–L divergence is not symmetric, we calculated the J–S divergence as the distance between the pair (*i,j*), defined as *JSD*(*P*^*i*^, *P*^*j*^) = (*D*_*KL*_(*P*^*i*^|*Q*_*ij*_) + *D*_*KL*_(*P*^*j*^|*Q*_*ij*_))/2, where the vector *Q*_*ij*_ = (*P*^*i*^ + *P*^*j*^)/2 represents the average distribution of microbiome community *i* and *j*. Based on the J–S divergence matrix, we also performed hierarchical clustering. Based on the distance matrix, we performed permutation tests to investigate whether the case group (cesarean birth or appendectomy) was significantly clustered relative to the whole set of samples.

### 2.5 Role of the Funding Source

The funding source had no direct role in study design, data collection, data analysis, interpretation, or writing the report.

## 3 Results

Of the 1097 participants, cesarean birth was reported as “yes” by 92, “no” by 948, and missing or uncertain by 57. Likewise, appendectomy was reported as “yes” by 155, “no” by 961, and missing or uncertain by 21. Selected characteristics of the participants and associations with cesarean birth and appendectomy history are presented in Table 1. The population in the cesarean birth analysis was 44% male, 93% non-Hispanic Caucasian, and distributed through all U.S. regions. They had a mean age of 46 [standard error (SE) 16] years and a mean BMI of 24 (SE 5). An omnivorous diet was reported by 80%, gluten sensitivity by 19%, and lactose sensitivity by 18%. Weight loss of at least 10 lb was reported by 10% and weight gain by 5%. An antibiotic had been used by 11% 2–6 months and 13% 7–12 months before participation. Cesarean birth was significantly associated with younger age, but not consistently with any of the other characteristics. History of appendectomy was significantly associated with older age and with higher BMI, and it tended to be more frequent in U.S. states outside California. Without adjusting for age or any other characteristic, cesarean birth and appendectomy histories had a weak inverse association (P = 0.11).

**Fig. 1:**
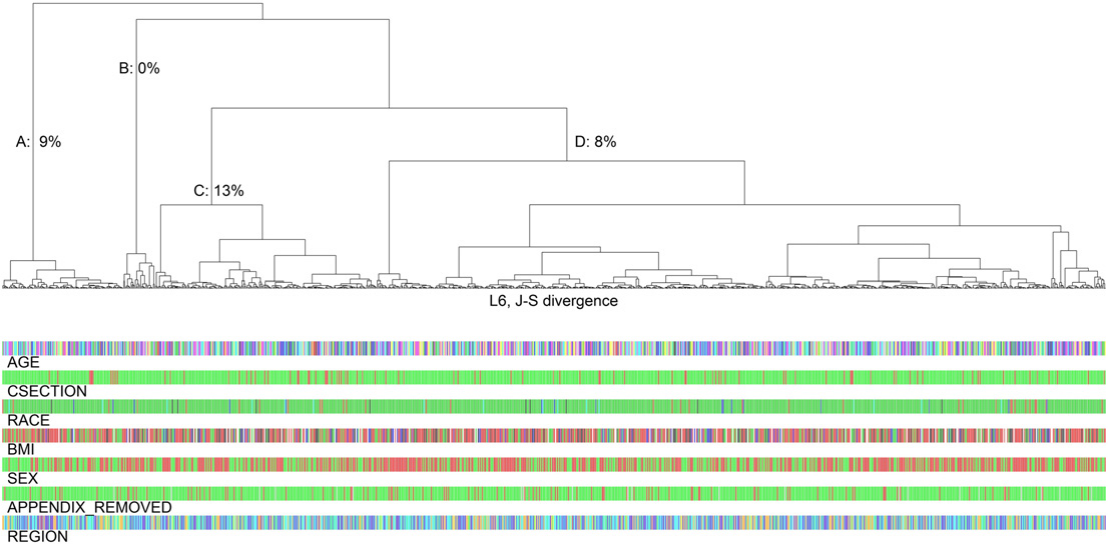
Dendogram of Jensen–Shannon (J–S) divergence and hierarchical clusters of 16S rRNA sequences at the L6 (genus) level for participants in the American Gut project. Each participant's category for age, history of cesarean birth (CSECTION), race/ethnicity, body mass index (BMI), sex, history of appendectomy, and geographic region is shown in colored bars at the bottom. The proportion of participants who reported cesarean birth was high in cluster C (28 of 209, 13%), low in cluster B (0 of 30, 0%), and intermediate in cluster A (10 of 114, 9%) and cluster D (54 of 689, 8%, P = 0.03).

In the 1097 fecal specimens, taxa were mapped to 261 bacterial families, 626 genera, and 844 species. Microbiome richness and alpha diversity estimates were not associated with cesarean birth or appendectomy history (Supplemental Table 1). In contrast, composition (beta diversity) of the fecal microbiome differed significantly for participants who reported cesarean birth. Average J–S divergence was strongly associated with cesarean birth at all taxonomic levels, from phylum (permutation P = 0.0076) to species (P = 0.0041, Table 2). In contrast, age was unrelated to average J–S divergence (R = 0.02). As shown in Fig. 1, of four genus-level J–S hierarchical clusters, the prevalence of cesarean birth was 9% in cluster A and 8% in cluster D, whereas cesarean prevalence was high in cluster C (13%) and low in cluster B (0 of 30, P = 0.03). Appendectomy history had no association with beta diversity defined by average J–S divergence (Table 2).

Table 3 presents 14 taxa with increased relative abundance and 3 taxa with decreased relative abundance with Cesarean birth history. Cesarean-delivered adults had nominally reduced abundance of *Coprobacillus* and *Holdemania* (phylum Firmicutes) and of *Neisseria* (Proteobacteria), as well as nominally increased abundance of five genera in the Lachnospiraceae, Peptococcaceae, and Ruminococcaceae families of Clostridia (Firmicutes), *Dysgonomonas* (Bacteroidetes, Bacteroidales) and *Salmonella* and *Haemophilus* (Proteobacteria, Enterobacteriaceae). Table 3 also shows the 22 taxa (14 Proteobacteria, 3 Actinobacteria, 3 Bacteroidetes, and 2 Firmicutes), all increased, with appendectomy history. These are nominally significant associations, none of which survived adjustment for multiple comparisons. Mean relative abundances for all 1949 taxa and Wald P-value comparisons are provided in Supplemental Table 2.

## 4 Discussion

This analysis was primarily motivated by the observation that the composition of the microbiome of neonates differed significantly between those born vaginally and those born by cesarean section (Arrieta et al., 2014, Dominguez-Bello et al., 2010). With vaginal delivery, the neonatal microbiome resembled the vaginal microbiome, with high relative abundance of *Prevotella* and especially *Lactobacillus* taxa. In contrast, cesarean-delivered neonates had a diverse array of taxa resembling the skin microbial community, including *Staphylococcus*, *Streptococcus*, Propionibacterineae, *Haemophilus*, and *Acinetobacter* (Dominguez-Bello et al., 2010). Cesarean-delivered neonates and infants typically have a paucity of *Bifidobacterium* and *Bacteroides* species (Arrieta et al., 2014). In the current analysis, we observed that the fecal microbiome composition differed in adults who reported that they had been delivered by cesarean section. This suggests that a difference by route of delivery may persist into adulthood. Of the taxa noted to be increased in cesarean-delivered neonates and infants (Arrieta et al., 2014, Penders et al., 2006, Dominguez-Bello et al., 2010), only *Haemophilus* and certain Clostridia genera had elevated abundance in the fecal microbiome of cesarean-delivered adults (Table 3).

In the placenta, microbiome composition (beta diversity) was reported to generally resemble the healthy oral microbiome. Placenta microbial composition differed for two types of women — those who reported a first-trimester urinary tract infection, with enrichment of several genera including *Streptococcus* and *Acinetobacter*; and those who had a pre-term delivery, with enrichment of *Burkholderia* and other genera (Aagaard et al., 2014). No overlap was observed between these taxa and the taxa that we found to differ, with nominal statistical significance, in the fecal microbiome of adults who had been born by cesarean section or who reported removal of their appendix (Table 3). The placenta microbiome was not found to differ between cesarean and vaginal birth (Aagaard et al., 2014).

Our study had several strengths, including relatively large size; high quality, unbiased profiles of the microbiome; restriction to individual adults; careful exclusion of recent antibiotic use and medical conditions that might have altered the microbiota; state-of-the-art statistical methods; and comparison of two conditions postulated to alter the gut microbiota. Although widely dispersed across the USA, the participants were not representative of the American adult population, being overwhelmingly non-Hispanic Caucasian (93%) and non-smokers (96%). Nonetheless, the prevalence of cesarean birth in our population, which was born on average in 1967, was 8.8%, comparable to the estimated cesarean rates of 5.5% and 10.4% for births throughout the USA in 1970 and 1975, respectively (Anon., 1995). The prevalence of appendectomy in our study, 14%, was similar to the 11%–14% prevalence reported by Dutch and British general population controls (Russel et al., 1997, Gent et al., 1994).

Our study also had important limitations, of which our lack of data on clinical indication for cesarean section may be the most important, given the fetal distress and antibiotic exposures associated with emergency cesarean. All of the covariate data (metadata) that we did have were self-reported, including birth and appendectomy history. We lacked data to confirm the Human Microbiome Project's finding that fecal microbial composition differed with self-reported history of having been breastfed (Ding and Schloss, 2014). The validity of some self-reported early life events is modest at best. Among Swedish adults, self-reported birth weight compared to birth records had a Spearman correlation of 0.76, and 53% of the self-reported birth weights were off by 250 g or more (Andersson et al., 2000). Among ethnically diverse, middle aged women in New York, self-report for pre-eclampsia in their mothers had a sensitivity of 36% but a specificity of 97% (Terry et al., 2009). Accuracy of self-report of cesarean birth is unknown. To reduce misclassification in our study, we excluded participants with uncertain history or missing data. We also excluded for self-reported use of an antibiotic within one month of participation. Nonetheless, altered taxonomy of the bacterial communities of both feces and saliva has been seen at 2 months to as long as 4 years after treatment with an antibiotic (Fouhy et al., 2012, Jakobsson et al., 2010, Dethlefsen and Relman, 2011). Despite this, both antibiotic use during infancy and cesarean birth, with or without antibiotic exposure, appear to increase the risk of obesity in childhood (Blustein et al., 2013, Trasande et al., 2013). In our study of adults, antibiotic use more than one month earlier was not associated with cesarean or appendectomy history (Table 1) and thus would not have substantially confounded our findings. Residual antibiotic effects or misclassification of birth history would likely bias toward the null. Finally, although we excluded duplicate participants, data were lacking to assess whether any twin pairs were included. This is an important limitation, because the composition and diversity of the gut microbiota within twin pairs is significantly more alike than expected by chance alone (Turnbaugh et al., 2009, Cozen et al., 2013).

In summary, we found a distinct difference in the composition of the fecal microbiota of adult volunteers who were born by cesarean section. History of appendectomy had no such distinction. The cesarean-birth distinction was independent of age and robust across all levels of bacterial taxonomy, but insufficient to define a cesarean-associated microbiome signature. The weaker association with hierarchical clusters (Fig. 1), suggests that many rare taxa contribute to the distinction. The individual taxa that were associated with cesarean birth or appendectomy (Table 3) were not statistically significant when adjusted for multiple comparisons, but they do provide hypotheses that can be examined in future studies. It remains to be seen whether neonatal differences in the gut microbiota directly or indirectly affect the risk of immunologic and metabolic diseases in adult life.

The following are the supplementary data related to this article.

Supplemental Table 1 Associations of microbiome richness and alpha diversity with cesarean birth or appendectomy history.

Supplemental Table 2 Associations of mean relative abundances for all 1949 taxa with cesarean birth or appendectomy history.

## Conflict of Interest

The authors wish to confirm that there are no known conflicts of interest associated with this publication and there has been no significant financial support for this work that could have influenced its outcome.

## Figures and Tables

**Table 1:** Selected characteristics of 1097 participants in the American Gut Project and associations with history of cesarean birth or appendectomy.

	Cesarean birth history (N = 92)				Appendectomy history (N = 115)			
Variable (N; N^a^)	Estimate	Std. error	z value	P-value⁎	Estimate	Std. error	z value	P-value⁎
Age, mean 46 years (SE 16 years)	− 0.04	0.01	− 6.11	9.7E− 10	0.05	0.01	6.51	7.5E− 11
Body mass index, mean 24 (SE 5) kg/m^2^	0.01	0.02	0.23	0.81	0.08	0.02	4.26	2.1E− 05
Sex, male (461; 486)	0.01	0.22	0.05	0.96	− 0.28	0.20	− 1.37	0.17
Gluten, yes (200; 204)	− 0.05	0.28	− 0.19	0.85	− 0.22	0.27	− 0.80	0.42
Lactose, yes (183; 187)	− 0.13	0.29	− 0.43	0.67	− 0.20	0.28	− 0.74	0.46
Asthma, yes (86; 89)	0.07	0.39	0.19	0.85	0.08	0.35	0.22	0.83

*Race/ethnicity*								
Caucasian, non-Hispanic (964; 995)	Referent				Referent			
African American (10; 10)	− 13.23	460.24	− 0.03	0.98	− 13.49	460.24	− 0.03	0.98
Hispanic (16; 16)	− 0.37	1.04	− 0.36	0.72	0.13	0.76	0.17	0.87
Asian or Pacific Islander (34; 38)	0.58	0.50	1.16	0.24	− 1.54	1.02	− 1.51	0.13
Other (16; 17)	− 0.37	1.04	− 0.36	0.72	− 0.70	1.04	− 0.67	0.50

*Geographic region*								
California (206; 213)	Referent				Referent			
Other western U.S. (224; 235)	0.04	0.36	0.10	0.92	− 0.64	0.30	− 2.12	0.03
Midwestern U.S. (164; 169)	− 0.06	0.40	− 0.16	0.87	− 0.60	0.33	− 1.80	0.07
Northeastern U.S. (196; 200)	0.35	0.35	1.02	0.31	− 0.36	0.30	− 1.21	0.23
Southern U.S. (210; 218)	0.33	0.34	0.96	0.34	− 0.56	0.30	− 1.85	0.06
Canada (22; 23)	0.63	0.67	0.93	0.35	− 0.16	0.65	− 0.25	0.80
Europe (11; 11)	− 14.09	723.49	− 0.02	0.98	− 0.57	1.07	− 0.53	0.59
Australia (6; 5)	− 14.09	979.61	− 0.01	0.99	1.33	0.93	1.42	0.15

*Antibiotic use*								
None in past year (793; 828)	Referent				Referent			
Yes, 2–6 months ago (112; 114)	− 0.25	0.39	− 0.63	0.53	0.48	0.29	1.67	0.10
Yes, 7–12 months ago (135; 134)	0.08	0.32	0.25	0.80	0.29	0.28	1.03	0.30

*Weight change*								
Remained stable (877; 908)	Referent				Referent			
Decreased > 10 lb (104; 107)	0.67	0.40	1.67	0.10	− 0.43	0.38	− 1.11	0.27
Increased > 10 lb (53; 54)	0.41	0.50	0.82	0.41	− 0.19	0.48	− 0.40	0.69

*Diet type*								
Omnivore (828; 856)	Referent				Referent			
No red meat (64; 68)	0.25	0.42	0.61	0.54	0.15	0.39	0.39	0.70
Vegan (29; 29)	0.19	0.62	0.31	0.76	0.01	0.62	0.01	0.99
Vegetarian (44; 47)	− 0.26	0.61	− 0.43	0.67	− 0.21	0.53	− 0.39	0.70
Pescetarian (71; 72)	0.14	0.42	0.33	0.74	0.56	0.34	1.66	0.10

*Alcohol consumption*								
Never (221; 226)	Referent				Referent			
Rarely (few times/month, 255; 265)	0.05	0.32	0.16	0.87	0.22	0.30	0.73	0.46
Occasionally (1–2 times/week, 214; 219)	0.20	0.33	0.60	0.55	− 0.13	0.34	− 0.40	0.69
Regularly (3–5 times/week, 205; 213)	0.08	0.34	0.24	0.81	0.55	0.30	1.83	0.07
Daily (142; 149)	− 0.33	0.42	− 0.78	0.43	0.01	0.36	0.03	0.97

*Tobacco smoking*								
Never (991; 1028)	Referent				Referent			
Rarely (few times/month, 25; 25)	1.01	0.51	1.96	0.05	− 0.32	0.74	− 0.43	0.67
More than rarely (17; 16)	0.85	0.65	1.32	0.19	− 0.59	1.04	− 0.57	0.57

a Number of participants, cesarean birth analysis; appendectomy analysis.

⁎ P-values by Wald test.

**Table 2:** K–L distance (beta diversity) analyses of cesarean birth and appendectomy history.

Taxonomic level	Test for clustering by self-reported history	
Cesarean birth	Appendectomy
P-value	P-value
Phylum	0.0076	0.79
Class	0.005	0.69
Order	0.004	0.66
Family	0.001	0.29
Genus	0.0018	0.29
Species	0.0041	0.32

**Table 3:** Individual taxa with nominally significant difference in relative abundance with history of cesarean delivery or appendectomy.

Cesarean-associated bacterial taxa	Wald P-value	Relative abundance Vaginal_Mean	Relative abundance Cesarean_Mean	Cesarean–vaginal difference
p__Actinobacteria;c__Coriobacteriia;o__Coriobacteriales;f__Coriobacteriaceae;g__Atopobium;s__rimae	0.01	4.22E− 07	2.17E− 06	1.75E− 06
p__Bacteroidetes;c__Bacteroidia;o__Bacteroidales;f__Porphyromonadaceae;g__Dysgonomonas;s__	0.01	1.52E− 05	5.76E− 05	4.24E− 05
p__Firmicutes	0.03	4.77E− 01	5.24E− 01	4.68E− 02
p__Firmicutes;c__Clostridia;o__Clostridiales	0.01	4.47E− 01	4.99E− 01	5.16E− 02
p__Firmicutes;c__Clostridia;o__Clostridiales;f__Lachnospiraceae	0.002	1.54E− 01	1.88E− 01	3.42E− 02
p__Firmicutes;c__Clostridia;o__Clostridiales;f__Lachnospiraceae;g__;s__	0.002	8.42E− 02	1.07E− 01	2.26E− 02
p__Firmicutes;c__Clostridia;o__Clostridiales;f__Lachnospiraceae;g__Roseburia	0.04	7.21E− 03	9.38E− 03	2.17E− 03
p__Firmicutes;c__Clostridia;o__Clostridiales;f__Peptococcaceae;g__rc4-4;s__	0.02	2.62E− 04	3.89E− 04	1.27E− 04
p__Firmicutes;c__Clostridia;o__Clostridiales;f__Ruminococcaceae	0.02	1.94E− 01	2.17E− 01	2.30E− 02
p__Firmicutes;c__Clostridia;o__Clostridiales;f__Ruminococcaceae;g__;s__	0.02	1.31E− 01	1.47E− 01	1.58E− 02
p__Firmicutes;c__Clostridia;o__Clostridiales;f__Ruminococcaceae;g__Ruminococcus;s__flavefaciens	0.03	7.95E− 04	1.45E− 03	6.51E− 04
p__Firmicutes;c__Erysipelotrichi;o__Erysipelotrichales;f__Erysipelotrichaceae;g__Coprobacillus;s__	0.03	1.88E− 04	5.33E− 05	− 1.35E− 04
p__Firmicutes;c__Erysipelotrichi;o__Erysipelotrichales;f__Erysipelotrichaceae;g__Holdemania;s__	0.05	3.76E− 04	2.62E− 04	− 1.14E− 04
p__Proteobacteria;c__Betaproteobacteria;o__Burkholderiales;f__Comamonadaceae;g__Methylibium	0.05	1.05E− 06	5.43E− 06	4.38E− 06
p__Proteobacteria;c__Betaproteobacteria;o__Neisseriales;f__Neisseriaceae;g__Neisseria;s__subflava	0.01	6.58E− 05	2.07E− 05	− 4.52E− 05
p__Proteobacteria;c__Gammaproteobacteria;o__Enterobacteriales;f__Enterobacteriaceae;g__Salmonella;s__enterica	0.01	4.22E− 07	3.26E− 06	2.84E− 06
p__Proteobacteria;c__Gammaproteobacteria;o__Pasteurellales;f__Pasteurellaceae;g__Haemophilus;s__influenzae	0.02	2.11E− 07	2.17E− 06	1.96E− 06

Appendectomy-associated bacterial taxa	Wald P-value	Vaginal_Mean	Cesarean_Mean	Cesarean–vaginal difference

p__Actinobacteria;c__Actinobacteria;o__Actinomycetales;f__Brevibacteriaceae;g__Brevibacterium;s__aureum	0.04	3.12E− 07	2.26E− 05	2.23E− 05
p__Actinobacteria;c__Actinobacteria;o__Actinomycetales;f__Micrococcaceae;g__Rothia;s__dentocariosa	0.03	3.23E− 06	9.57E− 06	6.34E− 06
p__Actinobacteria;c__Actinobacteria;o__Actinomycetales;f__Promicromonosporaceae;g__Cellulosimicrobium;s__	0.01	2.10E− 05	1.40E− 04	1.19E− 04
p__Bacteroidetes;c__Bacteroidia;o__Bacteroidales;f__;g__;s__	0.01	1.29E− 03	3.28E− 03	1.98E− 03
p__Bacteroidetes;c__Bacteroidia;o__Bacteroidales;f__Prevotellaceae;g__Prevotella;s__stercorea	0.02	7.72E− 04	2.44E− 03	1.67E− 03
p__Bacteroidetes;c__Flavobacteriia;o__Flavobacteriales;f__Flavobacteriaceae;g__Capnocytophaga;s__ochracea	0.04	2.08E− 07	8.70E− 07	6.61E− 07
p__Firmicutes;c__Clostridia;o__Clostridiales;f__Ruminococcaceae;g__Faecalibacterium	0.03	2.75E− 02	3.20E− 02	4.57E− 03
p__Firmicutes;c__Clostridia;o__Clostridiales;f__Ruminococcaceae;g__Faecalibacterium;s__prausnitzii	0.03	2.73E− 02	3.18E− 02	4.55E− 03
p__Proteobacteria;c__;o__;f__;g__;s__	0.03	1.87E− 06	6.96E− 06	5.08E− 06
p__Proteobacteria;c__Alphaproteobacteria;o__Sphingomonadales	0.05	1.02E− 04	4.33E− 04	3.31E− 04
p__Proteobacteria;c__Alphaproteobacteria;o__Sphingomonadales;f__Sphingomonadaceae	0.02	9.25E− 05	4.25E− 04	3.33E− 04
p__Proteobacteria;c__Alphaproteobacteria;o__Sphingomonadales;f__Sphingomonadaceae;g__Sphingobium;s__	0.02	4.80E− 05	2.29E− 04	1.81E− 04
p__Proteobacteria;c__Betaproteobacteria;o__Burkholderiales;f__Comamonadaceae;g__Acidovorax;s__	0.05	2.19E− 06	1.13E− 05	9.12E− 06
p__Proteobacteria;c__Betaproteobacteria;o__Nitrosomonadales;f__Nitrosomonadaceae;g__;s__	0.03	1.46E− 06	4.35E− 06	2.89E− 06
p__Proteobacteria;c__Gammaproteobacteria;o__Alteromonadales;f__[Chromatiaceae]	0.02	5.52E− 06	1.83E− 05	1.27E− 05
p__Proteobacteria;c__Gammaproteobacteria;o__Alteromonadales;f__[Chromatiaceae];g__;s__	0.03	4.99E− 06	1.74E− 05	1.24E− 05
p__Proteobacteria;c__Gammaproteobacteria;o__Alteromonadales;f__OM60	0.03	7.18E− 06	1.65E− 05	9.34E− 06
p__Proteobacteria;c__Gammaproteobacteria;o__Alteromonadales;f__OM60;g__;s__	0.05	6.87E− 06	1.57E− 05	8.78E− 06
p__Proteobacteria;c__Gammaproteobacteria;o__Enterobacteriales;f__Enterobacteriaceae;g__Morganella;s__	0.04	5.31E− 06	2.35E− 05	1.82E− 05
p__Proteobacteria;c__Gammaproteobacteria;o__Enterobacteriales;f__Enterobacteriaceae;g__Providencia	0.04	4.25E− 05	2.90E− 04	2.47E− 04
p__Proteobacteria;c__Gammaproteobacteria;o__Oceanospirillales;f__Halomonadaceae;g__Candidatus Portiera;s__	0.02	1.25E− 06	5.22E− 06	3.97E− 06
p__Proteobacteria;c__Gammaproteobacteria;o__PYR10d3;f__;g__;s__	0.03	1.21E− 05	2.43E− 05	1.23E− 05
